# Evaluation of small intestinal damage in a rat model of 6 Minutes cardiac arrest

**DOI:** 10.1186/s12871-018-0530-8

**Published:** 2018-06-05

**Authors:** Daniel C. Schroeder, Alexandra C. Maul, Esther Mahabir, Isabell Koxholt, Xiaowei Yan, Stephan A. Padosch, Holger Herff, Insa Bultmann-Mellin, Anja Sterner-Kock, Thorsten Annecke, Tim Hucho, Bernd W. Böttiger, Maria Guschlbauer

**Affiliations:** 1 Department of Anaesthesiology and Intensive Care Medicine, University Hospital of Cologne, Kerpener Str. 62, Cologne, Germany; 2 Experimental Medicine, University Hospital of Cologne, Robert-Koch-Str.10, Cologne, Germany; 3 Comparative Medicine, Center for Molecular Medicine Cologne, University of Cologne, Robert-Koch-Str.21, Cologne, Germany; 4 Decentral Animal Facility, University Hospital of Cologne, Robert-Koch-Str.10, Cologne, Germany

**Keywords:** Cardiac arrest, Small intestine, Ischaemia-reperfusion-injury, Systemic inflammatory response syndrome

## Abstract

**Background:**

Contribution of the small intestine to systemic inflammation after cardiac arrest (CA) is poorly understood. The objective was to evaluate whether an in vivo rat model of 6 min CA is suitable to initiate intestinal ischaemia-reperfusion-injury and to evaluate histomorphological changes and inflammatory processes in the small intestinal mucosa resp. in sera.

**Methods:**

Adult male Wistar rats were subjected to CA followed by cardio-pulmonary resuscitation. Proximal jejunum and serum was collected at 6 h, 24 h, 72 h and 7 d post return of spontaneous circulation (ROSC) and from a control group. The small intestine was evaluated histomorphologically. Cytokine concentrations were measured in jejunum lysates and sera.

**Results:**

Histomorphological evaluation revealed a significant increase in mucosal damage in the jejunum at all timepoints compared to controls (*p* < 0.0001). In jejunal tissues, concentrations of IL-1α, IL-1β, IL-10, and TNF-α showed significant peaks at 24 h and were 1.5- to 5.7-fold higher than concentrations at 6 h and in the controls (*p* < 0.05). In serum, a significant higher amount of cytokine was detected only for IL-1β at 24 h post-ROSC compared to controls (15.78 vs. 9.76 pg/ml).

**Conclusion:**

CA resulted in mild small intestinal tissue damage but not in systemic inflammation. A rat model of 6 min CA is not capable to comprehensively mimic a post cardiac arrest syndrome (PCAS). Whether there is a vital influence of the intestine on the PCAS still remains unclear.

**Electronic supplementary material:**

The online version of this article (10.1186/s12871-018-0530-8) contains supplementary material, which is available to authorized users.

## Background

Cardiac arrest (CA) results in transient systemic ischaemia followed by reperfusion as a consequence of successful resuscitation. First, cessation of circulation results in depletion of oxygen followed by ATP-dependent Na^+^/K^+^-pump dysfunction. Resulting in a breakdown of cellular integrity, glutamate is being released intracellularly and mediates cerebral excitotoxicity by activation of N-methyl-D-aspartate receptors. Subsequently, intracellular Ca^++^ influx and activation of caspases, phospholipases, and proteases lead to cellular death [1]. Secondly, subsequent reperfusion triggers formation of free radicals, which further aggravates cell death. Later, accumulation of inflammatory cytokines is initiated and causes a long-lasting inflammatory reaction.

Within 4–5 min after CA, selectively vulnerable regions in the brain such as the hippocampal CA1 become apoptotic and necrotic [2]. As a result, brain injury is responsible for the mortality of 68% of the victims of CA [3]; neurocognitive long-term impairment occurs in half of the survivors [4]. In addition to that, within hours to days, the characteristic systemic ischaemia-reperfusion-injury provokes a systemic inflammatory release also known as sepsis-like- or post-cardiac arrest syndrome (PCAS) ending up in multiple organ failure [5, 6]. In fact, CA is a complex systemic ischaemia-reperfusion-injury with contribution of multiple independent tissue. However, to date, particular contribution of peripheral organs to the development of PCAS is not clearly understood. Given that more than 30% of victims of CA show bacteremia upon presentation, the small intestine was discussed to be an immediate by-product of a systemic ischaemia-reperfusion-injury [7, 8].

As described, the small intestine is highly susceptible to a focal ischaemia-reperfusion-injury [9, 10]. Already after 15–30 min of mesenterial occlusion, morphological changes such as atrophy of the villi and damage of tunica mucosa and tunica serosa appear [11]. Consequently, loss of intestinal integrity is associated with excessive fluid loss and translocation of gut bacteria and toxins into the blood circulation [12]. Subsequent restoration of blood flow leads to an activation of molecular and cellular components of the innate immunity resulting in an inflammatory response [13]. As a result, local and systemic inflammation occurs and causes a multiple organ dysfunction syndrome with a mortality rate reported between 30–90% [9, 10, 13–15].

However, due to the predominant brain damage decisive for morbidity and mortality in survivors of CA the role of the small intestine in the development of systemic inflammation after CA was not intensively investigated yet. Thus, the objective was (i) to evaluate whether an in vivo rat model of 6 min CA is suitable to initiate an intestinal ischaemia-reperfusion-injury to further examine (ii) genesis of local and systemic inflammation. It was hypothesized that mild small intestinal damage occurs even after short durations of CA and resuscitation.

## Methods

### Animals and husbandry

Seventy-eight 7 to 8 weeks old male Wistar rats (Janvier, France) weighing 280 - 320 g were transferred into the animal facility 10 days before surgery and had ad libitum access to standard pelleted feed (Ssniff®, V1534–703, Germany) and water. Rats were housed under a 12:12 h light-dark cycle at 22 °C and a relative humidity of 60%. They were allocated randomly to 5 groups (controls, 6 h, 24 h, 72 h and 7 d post-ROSC).

### Cardiac arrest and cardio-pulmonary resuscitation

The detailed experimental protocol was previously published by Böttiger et al. [16]. Briefly, rats were anesthetized with 3% sevoflurane and 70% nitrous oxide in oxygen. Animals were endotracheal intubated (Braunüle-MT No. 3, Braun, Germany) and ventilated at a rate of 60 breaths per min (Rodent Ventilator, Harvard Apparatus, MA, USA). A saline-filled polyethylene catheter was advanced via cut-down into the left femoral artery to continuously measure mean arterial pressure (MAP, SC7000, Siemens Health Care GmbH, Germany). Another saline-filled polyethylene catheter was advanced via cut-down into the left femoral vein for drug administration. The tidal breathing volume was adjusted to ensure a physiological pCO_2_ (35-45 mmHg). The inspired oxygen concentration (FiO_2_) was regulated to ensure a physiological pO_2_. Blood gas analysis was performed using ABLFlex800 (Radiometer, Germany). The cardio-pulmonary resuscitation (CPR) protocol fulfils the Utstein Style guidelines for laboratory CPR research [17]. The rats received an oesophageal electrode for induction of ventricular fibrillation (12 V, 50 Hz, 1.5 min) until the mean arterial blood pressure stayed below 15 mmHg [18]. After 5.5 min of CA, rats were mechanically ventilated using 100% oxygen at 60 breaths per min. At 6 min after CA, CPR started by performing a manual closed-chest cardiac massage (200 times/min) and an injection of 20 μg/kg epinephrine (Suprarenin, Sanofi-Aventis, Germany). Two min later, a single bi-phasic shock of 2–3 J (M series, Zoll Corporation, Germany) was attempted. Epinephrine administration and biphasic shocks were repeated every 30 s. ROSC was defined as maintenance of mean arterial blood pressure above 50 mmHg for at least 10 min. If ROSC was not achieved after 6 min of CPR, resuscitation procedures were terminated. To maintain normocapnia, ventilation rate was adjusted and sodium bicarbonate was titrated according to the blood gas analysis. Once adequate spontaneous breathing was observed, rats were extubated, kept singly and monitored every 2–4 h.

### Euthanasia and tissue sampling

A total of 55 rats (70.5%) could not be resuscitated. All 23 successfully resuscitated and sham-operated rats survived the observation period and were included in the study. The number of rats used for histomorphological analysis and cytokine profiling is shown in Table 1. One group was used as sham-operated control-group and was euthanized immediately after surgical procedures without CA and CPR. Under anesthesia, the thorax was opened and blood samples were taken from the left ventricle. Following coagulation for 45 min at room temperature, the tubes (Eppendorf, Germany) were centrifuged for 10 min at 2500 g and 4 °*C. sera* were aliquoted and stored at − 80 °C. A 2–3 cm piece of the mid jejunum, 8 cm distal from the pylorus, was excised and divided. One segment was shock-frozen in liquid nitrogen and stored at − 80 °C for the multiplex cytokine assay. The other segment was fixed in 4% formalin for histomorphological studies.

**Table 1:** Number of rats used for histomorphological analysis (HA) and cytokine profiling (CP) of controls (C) and at 6 h, 24 h, 72 h and 7 d post-ROSC

	Study groups and number of rats				
Analysis	C	6 h	24 h	72 h	7 d
Jejunum HA	4	3	5	4	4
Jejunum CP	4	3	5	6	4
Serum CP	6	3	6	4	4

This analysis is a sub-study of an investigation aimed to pursue systemic inflammation in multiple tissues after CA. Serum cytokine profiling was conducted in *n* = 23 rats. Only *n* = 3 animals were resuscitated and included in the 6 h group. At least *n* = 4 animals were included in the 24 h, 72 h and 7 d group. Additionally, *n* = 2 more sera in the control group and 1 more serum sample in the 24 h group were collected and analyzed from further experiments. In the 72 h group, n = 2 more jejunal samples were also analyzed

### Histomorphological analysis - Chiu grading

Paraffin-embedded jejunal tissue was sectioned (4 μm) and stained with hematoxylin and eosin (H&E) according to standard protocols. The morphological integrity of the intestinal wall was classified by a blinded investigator using a modified protocol according to Chiu et al. [19]: Grade 0: normal mucosa; Grade 1: development of a sub-epithelial space at the tips of the villi; Grade 2: more extended sub-epithelial space at the tips of the villi, development of Gruenhagen’s space at the tips of the villi; Grade 3: massive epithelial lifting down the sides of the villi, villus necrosis; Grade 4: villi are denuded of epithelial layer; Grade 5: loss of villi, mucosal ulceration and necrosis with invasion of the muscularis propria. To evaluate oedema formation within the jejunal wall, the thickness of serosa, muscularis, submucosa and mucosa was measured using 10-fold magnifications (Olympus DP25, cellSens Standard 1.11, Olympus GmbH, Germany).

### Tissue lysates and protein extraction

Proteins were extracted by homogenizing 200 mg jejunal tissue with 500 μl RIPA buffer (150 mM NaCl, 1% Triton-x-100, 1% Na-deoxycholate, 0.1% SDS, 50 mM Tris-HCl pH 8, 10 mM EDTA) containing a proteinase-inhibitor (complete Mini, EDTA-free, Roche, Switzerland). Subsequently, the tissue lysates were centrifuged for 15 min at 11000 g and 4 °C and supernatants were centrifuged for 40 min at 44,400 g and 4 °C. The clear supernatants were analyzed using the BCA test (Pierce, Thermo Scientific, Germany) and duplicate aliquots of 1000 μg/ml protein were measured directly.

### Multiplex cytokine assay

IL-1α, IL-1β, IL-6, IL-10 and TNF-α in jejunal tissues and IL-1α, IL-1β, IL-2, IL-4, IL-5, IL-6, IL-10, IL-12 (p70), IL-13, interferon-γ (IFN-γ), granulocyte macrophage colony-stimulating factor (GM-CSF) and TNF-α in serum were measured in duplicates in a multiplex analyzer (Bio-Plex 200®, Bio-Rad Laboratories, USA) according to the manufacturer’s instructions. The jejunal tissue lysate protein was diluted 1:2 with sample diluent containing 0.5% BSA (500 μg/ml final protein concentration). Sera were thawed, centrifuged for 5 min at 10,000 g and 4 °C and diluted 1:4 in sample diluent. By using the median fluorescence intensity and the standard curve, the absolute concentration of each cytokine (pg/ml) was calculated (Bio-Plex Manager 6.1, Bio-Rad Laboratories). The value 0 was attributed when the results were below the detection limit.

### Statistical analyses

An a priori power analysis was performed to determine the adequate sample size for detection of TNF-α in serum 24 h after CA as primary outcome variable. (type 1 error: 5% (α < 0.05); type 2 error: 20% (β < 0.80); medium efficiency: 0.6). Animal studies reporting serum TNF-α concentrations after CA due to ventricular fibrillation are scarce. According to the literature, an average rise from 0 to approximately 12 pg/mL [5] in humans is expected. Since no serum TNF-α was expected on day 0 (controls), a variability of 0 was expected. To display a difference in serum TNF-α concentrations, we estimated a number of 4 rats per group.

One-way ANOVA and Tukey’s multiple comparison test was performed using GraphPad Prism 6 for Windows. For correlation of the duration of CA with cytokine concentrations and Chiu-grades, the Pearson’s correlation test was used with unilateral values (GraphPad Software, USA). All data are presented as mean ± SD. A *p*-value< 0.05 was considered statistically significant.

### Ethical statement

All procedures were ethically approved by the appropriate governmental authority (Landesamt für Natur, Umwelt und Verbraucherschutz Nordrhein-Westfalen, LANUV, Germany, AZ: 8.87–51.04.20.09.368) and were in accordance with the German Animal Welfare Law. Animal care and use was performed by qualified individuals, supervised by a veterinarian. All facilities and transportation complied with current legal requirements. The manuscript complies with the Animals in Research: Reporting In Vivo Experiments (ARRIVE) guidelines [20]. Humane endpoints were specified and part of the animal welfare application according to the directive 2010/63/EU of the European parliament. A list of specific clinical signs to determine the animal’s physiology and behavioral condition was used. Specific experiment-related humane endpoints such as neurological disturbances, lameness, wound healing deficits and coma were evaluated. The animals were scored as mild, moderate or severely impaired. According to the results of scoring, animals were treated (analgesics, antibiotics), frequently examined or sacrificed (severe impairment). According to the results of the scored humane endpoints, animals received 50 μg/ml meloxicam p.o. within the first three days after ROSC, if necessary.

## Results

### Histomorphological analysis - Chiu grading

**Fig. 1:**
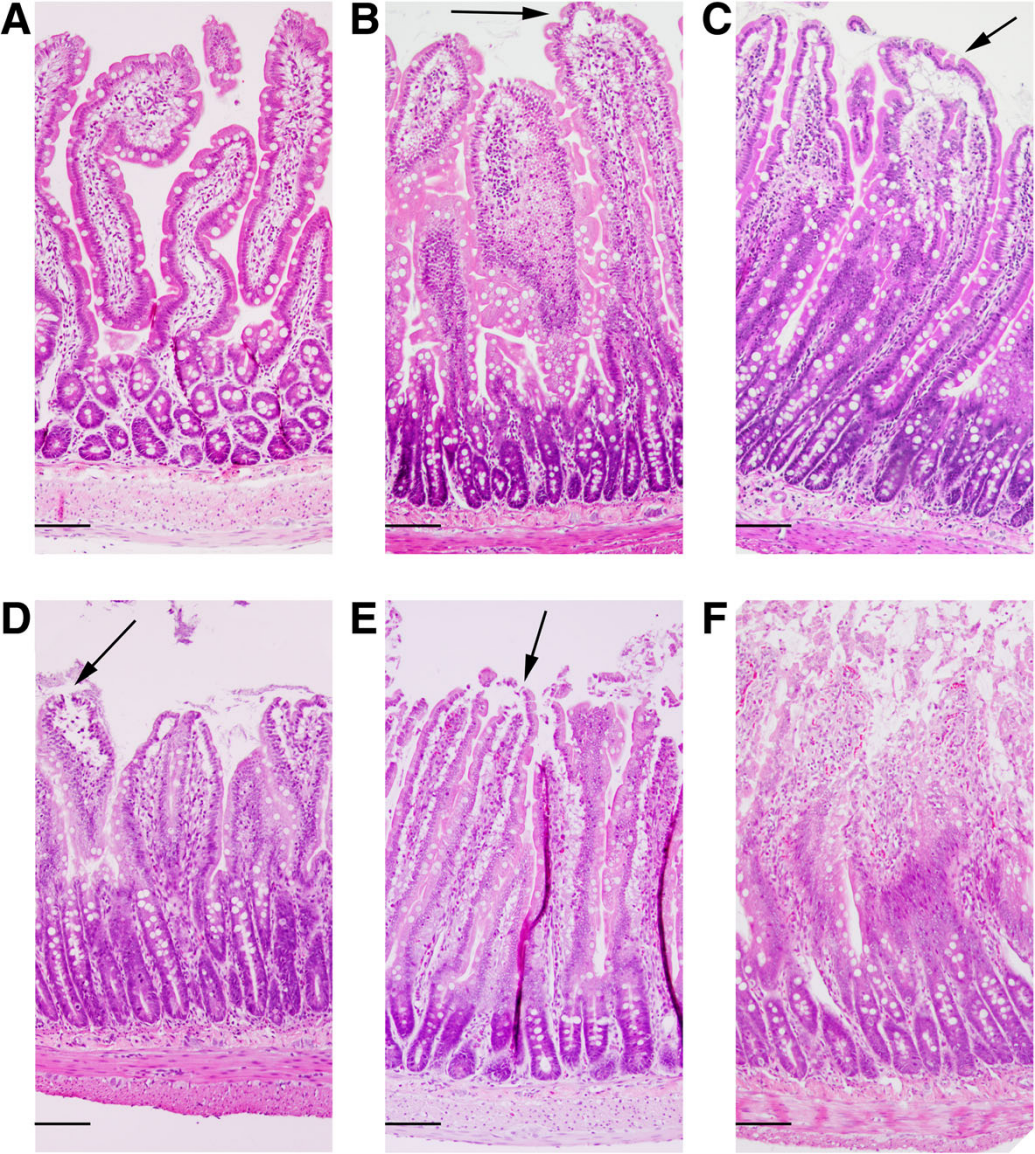
Histomorphological evaluation of the jejunal wall of rats after 6 min of CA using a modified Chiu scoring system (Chiu et al. 1970) [44]. Images represent the Chiu grades 0–5. **a** Intact mucosa and villus structures in control tissue of sham-operated rats (Chiu grade 0). **b** Development of a sub-epithelial space at the tips of the villi (arrow) 24 h post-ROSC (Chiu grade 1). **c** Development of a Gruenhagen’s space at the tip of a villus (arrow) 24 h post-ROSC (Chiu grade 2). **d** Villus necrosis (arrow) 7 d post-ROSC (Chiu grade 3). **e** Massive epithelial desquamation and villi which are denuded of epithelial layer (arrow) 7 d post-ROSC (Chiu grade 4). **f** Loss of villi, mucosal ulceration and necrosis 7 d post-ROSC (Chiu grade 5). **a**-**f** Hematoxilin-eosin staining, longitudinal section of 4 μm thickness (**a**, **e**, **f**) or cross section (**b**, **c**, **d**), scale bars represent 100 μm

**Fig. 2:**
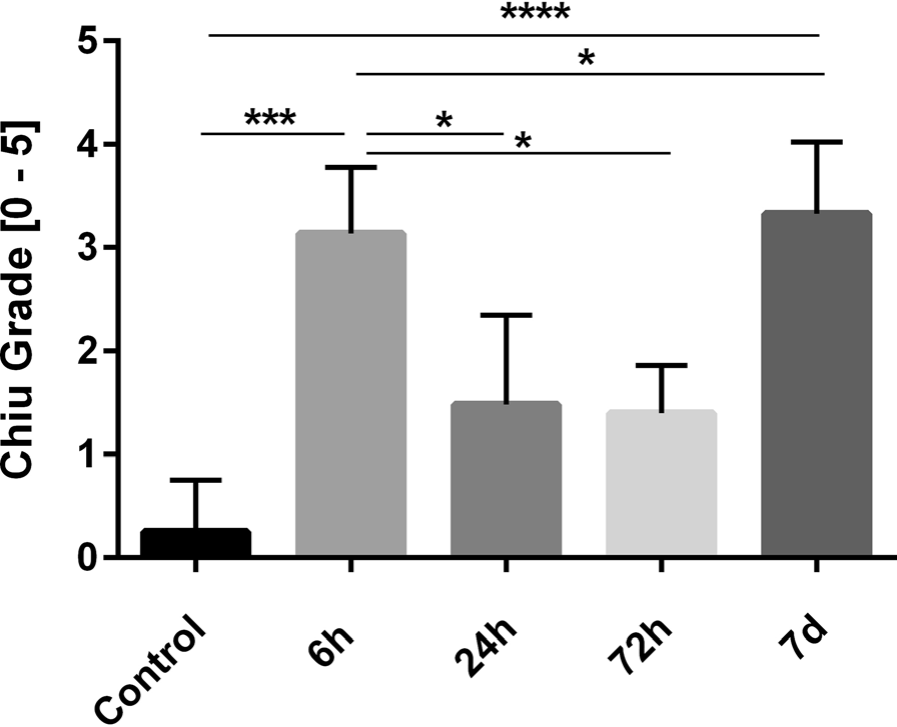
Chiu Scoring. Chiu Scoring [Grades 0–5] in jejunum of controls and at 6 h, 24 h, 72 h and 7 d post-ROSC are expressed as mean ± SD. One-way ANOVA *p* < 0.0001. Tukey’s multiple comparison test is marked with asterisks (*p < 0.05, ***p < 0.001, ****p < 0.0001)

Representative images of intestinal tissues after CA revealing Chiu grades from 0 to 5 are shown in Fig. 1a-f. Controls lacked histomorphological changes in the small intestine. In rats with 6 min of CA, the intestinal mucosa revealed desquamation of the villus tips. Blunt, dome-shaped, fenestrated epithelial cells of submucosal arterioles were evident. Gruenhagen’s spaces, slight perivascular oedema, hydropic generation of epithelial cells and sparse pyknotic cells were identified. Chiu grades of jejunal mucosa revealed a significant time-dependent effect (Fig. 2, *p* < 0.0001). Tukey’s multiple comparison test showed a significant increase (12.5-fold) in mucosal damage 6 h post-ROSC (3.13 ± 0.64) compared to controls (0.25 ± 0.5, *p* < 0.001). Mucosal damage decreased significantly 2.1-fold at 24 h (1.48 ± 0.87) compared to 6 h post-ROSC (3.13 ± 0.64, *p* < 0.05). Chiu grading decreased significantly 2.2-fold at 72 h (1.4 ± 0.46) compared to 6 h post-ROSC (*p* < 0.05). At 7 d post-ROSC, Chiu grade increased again (3.33 ± 0.69, *p* < 0.05) compared to 24 h (1.48 ± 0.87) and was the highest compared to controls (0.25 ± 0.5, *p* < 0.0001).

Comparing the intestinal wall thickness of controls to that at 6 h, 24 h, 72 h and 7 d post-ROSC showed significant time-dependent changes (*p* < 0.0001). Tukey’s multiple comparison test showed that the muscularis was significantly thinner at 7 d post-ROSC compared to controls (*p* < 0.05). A thinner muscularis was also observed 6 h, 24 h and 72 h post-ROSC but this difference was not significantly decreased compared to controls.

### Multiplex cytokine assay

#### Jejunal tissue

**Fig. 3:**
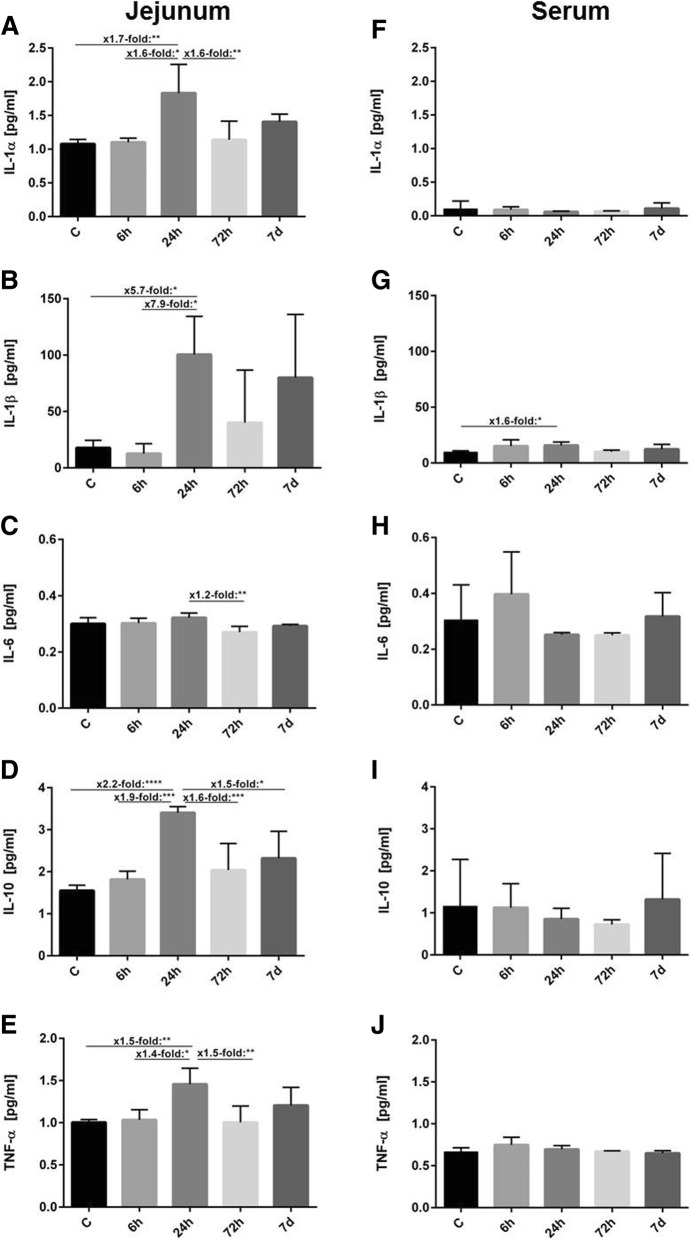
Cytokine concentrations of IL-1α, IL-1β, IL-6, IL-10 and TNF-α (mean ± SD, pg/ml) in jejunum (left column) and serum (right column) in controls and at 6 h, 24 h, 72 h and 7 d post-ROSC. The significance of Tukey’s multiple comparison test is marked with asterisks (*p < 0.05, **p < 0.01, ***p < 0.001, ****p < 0.0001). **a** IL-1α concentrations in jejunum (one-way ANOVA *p* < 0.01). **b** IL-1β concentrations in jejunum (one-way ANOVA *p* < 0.01). **c** IL-6 concentrations in jejunum (one-way ANOVA *p* < 0.01). **d** IL-10 concentrations in jejunum (one-way ANOVA *p* < 0.0001). **e** TNF-α concentrations in jejunum (one-way ANOVA *p* < 0.01). **f** IL-1α concentrations in serum (one-way ANOVA *p* > 0.05). **g** IL-1β concentrations in serum (one-way ANOVA*). **h** IL-6 concentrations in serum (one-way ANOVA *p* > 0.05). **i** IL-10 concentrations in serum (one-way ANOVA *p* > 0.05). **j** TNF-α concentrations in serum (one-way ANOVA *p* > 0.05)

In jejunal tissue, IL-1α was altered over time with peak values at 24 h post-ROSC. (*p* < 0.01, Fig. 3a). Tukey’s multiple comparison test identified significant elevations in IL-1α at 24 h compared to controls (1.7-fold increase, *p* < 0.01,) and the 6 h group (1.7-fold increase, *p* < 0.05). At 72 h, IL-1α concentration decreased significantly 1.6-fold (*p* < 0.01) compared to the 24 h group. No significant difference was detected between 24 h and 7 d post-ROSC (Fig. 3a). IL-1β showed significant time-dependent changes (*p* < 0.01). IL-1β peaked at 24 h compared to controls (5.7-fold increase, *p* < 0.05) and 6 h (7.9-fold increase, *p* < 0.05). At 72 h, IL-1β was 2.5-fold lower than at 24 h and 1.9-fold lower than at 7 d post-ROSC but differences were not statistically different (Fig. 3b). IL-6 concentrations showed time-dependent changes (*p* < 0.01). Additionally, a significant 1.2-fold decrease was detected at 72 h compared to 24 h post-ROSC (*p* < 0.01, Fig. 3c). IL-10 concentrations revealed significant time-dependent changes (*p* < 0.0001). At 24 h, it peaked significantly compared to controls (*p* < 0.0001) and 6 h post-ROSC (*p* < 0.001) resulting in a 2.2-fold and 1.9-fold increase, respectively. Concentrations at 72 h (1.6-fold, *p* < 0.001) and 7 d (1.5-fold, *p* < 0.05) decreased significantly compared to that at 24 h (Fig. 3d). TNF-α concentrations showed a significant time-dependent effect (*p* < 0.01) at 24 h post-ROSC compared to controls (1.5-fold increase, *p* < 0.01) and 6 h post-ROSC (1.4-fold increase, *p* < 0.05). TNF-α decreased significantly at 72 h post-ROSC compared to 24 h post-ROSC (1.5-fold, *p* < 0.01). There were no significant changes between 72 h and 7 d post-ROSC (Fig. 3e).

#### Serum

Absolute concentrations of IL-2, IL-4, IL-5, IL-12 (p70), IL-13, GM-CSF and IFN-γ in serum are shown in Table 2. Results for IL-1α, IL-1β, IL-6, IL-10 and TNF-α are shown in Fig. 3f-j. Serum concentrations of IL-1α, IL-2, IL-4, IL-5, IL-6, IL-10, IL-12 (p70), IL-13, GM-CSF, IFN-γ and TNF-α did not differ significantly between controls and at 6 h, 24 h, 72 h and 7 d post-ROSC. IL-1β showed a significant time-dependent effect (*p* < 0.05). At 24 h post-ROSC, IL-1β significantly increased 1.6-fold compared to controls (*p* < 0.05, Fig. 3g).

**Table 2:** Absolute levels of cytokines in serum (IL-2, IL-4, IL-5, IL-12 (p70), IL-13, GM-CSF, IFN-γ) in controls (C) and at 6 h, 24 h, 72 h and 7d post-ROSC expressed in pg/ml (mean ± SD)

	C	6 h	24 h	72 h	7d
IL-2	0.36 ± 0.35	0.27 ± 0.06	0.20 ± 0.05	0.21 ± 0.07	0.34 ± 0.28
IL-4	0.24 ± 0.18	0.22 ± 0.09	0.17 ± 0.02	0.17 ± 0.02	0.23 ± 0.10
IL-5	n.d.	n.d.	n.d.	n.d.	n.d.
IL-12 (p70)	n.d.	n.d.	n.d.	n.d.	n.d.
IL-13	n.d.	n.d.	n.d.	n.d.	n.d.
GM-CSF	0.20 ± 0.01	0.25 ± 0.06	0.23 ± 0.02	0.24 ± 0.03	0.23 ± 0.05
IFN-γ	0.36 ± 0.07	0.47 ± 0.18	0.34 ± 0.01	0.31 ± 0.02	0.35 ± 0.05

*n.d.* not detected

### Comparison of jejunum and serum cytokine concentrations

The ratio of absolute cytokine concentrations in jejunum compared to serum is shown in Table 3. Overall, significant higher IL-1α and TNF-α concentrations were observed in jejunal tissue compared to serum in both controls and CA groups at all timepoints (*p* < 0.05). IL-1β in controls and at 24 h post-ROSC, IL-6 at 24 h post-ROSC and IL-10 at 24 h and 72 h post-ROSC were also significantly higher in jejunum lysates compared to serum. Increased concentrations of IL-1β at 72 h and 7 d post-ROSC and IL-10 in controls and at 6 h and 7 d post-ROSC were detected in jejunum but were not significantly different. IL-6 concentrations in jejunum and serum in controls and at 6 h, 72 h and 7 d post-ROSC and IL-1β concentrations at 6 h post-ROSC were comparable. Comparing the time-dependent profiles of jejunum/serum ratio with profiles in jejunal tissue, IL-1α, IL-6, IL-10 and TNF-α displayed a similar alteration with a peak at 24 h post-ROSC while IL-1β peaked at 7 d post-ROSC, in contrast to jejunal tissue at 24 h post-ROSC (Fig. 3a-e).

**Table 3:** Ratio of absolute IL-1α, IL-1β, IL-6, IL-10, TNF-α levels in jejunum to absolute levels in serum in controls (C) and at 6 h, 24 h, 72 h and 7d post-ROSC (mean ± SD)

	C	6 h	24 h	72 h	7d
IL-1α	15.2 ± 3.6^****^	15.5 ± 1.7^****^	30.4 ± 3.8^****^	19.3 ± 4^***^	13.9 ± 5^****^
IL-1β	1.7 ± 0.2^*^	1 ± 0.6	6.9 ± 0.4^**^	7.9 ± 2.9	10.2 ± 1.5
IL-6	1 ± 0.1	1 ± 0.04	1.3 ± 0.03^****^	1 ± 0.02	1.1 ± 0.1
IL-10	1.9 ± 0.4	1.8 ± 0.3	4.2 ± 0.6^****^	3.6 ± 0.5^*^	2.5 ± 0.7
TNF-α	1.5 ± 0.04^****^	1.4 ± 0.1^*^	2.1 ± 0.1^***^	1.7 ± 0.2^*^	2 ± 0.1^**^

The level of significance is marked with asterisks: *p < 0.05, **p < 0.01 ***p < 0.001, ****p < 0.0001)

### Correlation analysis

No significant correlation between the duration of CA and cytokine concentrations in jejunum was detected 6 h and 24 h post-ROSC: 6 h group: IL-1α: *r* = 0.43, *p* = 0.546; IL-1β: *r* = 0.002, *p* = 0.970; IL-6: *r* = 0.06, *p* = 0.848; IL-10: *r* = 0.44, *p* = 0.532; TNF-α: *r* = 0.07, *p* = 0.824. 24 h group: IL-1α: *r* = 0.01, *p* = 0.874; IL-1β: *r* = 0.26, *p* = 0.492; IL-6: *r* = 0.86, *p* = 0.073; IL-10: *r* = 0.34, *p* = 0.413; TNF-α: *r* = 0.32, *p* = 0.438. However, there was a statistically significant correlation between the duration of CA and the Chiu-Grade 7 d after CA (*r* = 0.97, *p* = 0.012), while the other timepoints were not statistically significant (6 h group: *r* = 0.85, *p* = 0.251; 24 h group: *r* = 0.24, *p* = 0.501; 72 h group: *r* = 0.21, *p* = 0.537). Additional information pertaining to the duration of cardiac arrest and the different groups are shown in Additional file 1 but no significant differences were found between groups.

## Discussion

This study reveals three major findings: (i) mild intestinal barrier damage could be detected within 24 h in a rat model of 6 min CA. (ii) only mild local intestinal inflammation could be shown within 24 h after CA. (iii) a systemic inflammation and thus a potential contribution of the small intestine to systemic inflammation could not be simulated after 6 min of CA in rats.

### Intestinal barrier damage

Although 6 min of transient global ischaemia and subsequent reperfusion led to mucosal damage and decreased thickness of the muscularis in the small intestine, jejunal tissue concentrations of IL-1α, IL-1β, IL-10 and TNF-α were only slightly affected. This result may be due to several reasons.

First, the duration of 6 min CA in the present study that is sufficient to show cerebral damage [21] is inadequate to initiate a systemic inflammatory response, which is a clear limitation of this study. As shown by Qian, exceeding the duration of CA of 6 min may extend intestinal damage and influence serum cytokine concentrations. In detail, intestinal microcirculatory blood flow was significantly decreased accompanied by mild elevated serum concentrations of TNF-α and IL-6 during 8 min of CA in pigs [22]. In contrast, significant inflammatory response following local ischaemia and reperfusion of the small intestine was shown to be initiated after a duration of at least 15–30 min [23]. However, models of focal ischaemia and reperfusion are not capable of being translated to conditions of systemic ischaemia and reperfusion. In fact, CA leads to a complex systemic ischaemia-reperfusion-injury with contribution of multiple independent tissues, which are integrated into a complicated cascade of cell-death and systemic inflammation [5, 6].

Secondly, intestinal repair mechanisms seem to be commenced immediately after CA. A normalization of leukocyte-endothelial interaction as well as the wall shear rate was reported to be initiated within 120 min after CA, which is a period not mirrored in our study [24]. However, reports show that cytokines such as IL-1ra, IL-6, IL-8, IL-10 and TNF-α are expressed within 3 h [5, 6, 25] to 6 h [24, 26] and peak within the first 2 days after CA [5, 6]. This is in line with studies reporting a massive up-regulation of cytokines after ischaemic brain injury [2, 27]. Due to oxidative stress, NF-КB is up-regulated and orchestrates the release of a number of cytokines such as IL-1ß, IL-2, IL-4, IL-5, IL-6 and IL-10, IL-12, IL-13 and TNF-α [28, 29]. Accordingly, we collected blood (sera) and tissues at early stages including 6 h and 24 h post-ROSC and chose a similar cytokine profile to be investigated. However, in clinical studies, it is often proposed that the PCAS may result from a systemic inflammatory activation persisting for days [5, 30]. Therefore, cytokine concentrations in jejunum and sera were also evaluated within the first 7 days after CA in the present study.

Third, as repeatedly shown, simulation of a PCAS is difficult in rats and accompanied by a high failure rate of more than 50% [24, 31]. Thus, on the basis of previous scientific findings, we conclude, that severely injured rats that would develop a PCAS initially died during resuscitation procedures. With a survival rate of only 29.5% in the present study, we conclude that this model can be utilized to a limited extent to reproduce a PCAS or peripheral tissue damage, respectively. Nevertheless, ROSC rates are comparable to previous experiments performed in our group, which are able to adequately determine cerebral damage [16, 18, 21, 24, 32–34]. In this pilot study, our main objective was to evaluate peripheral tissue potentially injured due to CA. Notably, we did not lose any animals after ROSC, which is advantageous since post-ROSC mortality is known to be 38% within an observation period > 48 h [35]. Interestingly, Vognsen et al. recently showed that only 12% of animal studies sufficiently report outcome parameters according to the Utstein Guidelines, which was a strong criterion to increase the validity of this study [35].

Given the clinical phenomenon of bacteremia after CA [7, 8], intestinal damage seems to be conclusive even after short periods of ischaemia. Congruently, our results point towards a mild local intestinal damage, which is in line with Pan et al. who reported similar Chiu scorings 24 h after CA of 6 min duration [36]. Likewise, Teschendorf et al. showed a 3–4-fold stronger plasma extravasation from post-capillary mesenteric venules at 120 min after CA, which is a characteristic sign of endotoxaemia [24]. Another factor taken into consideration is the short initial tissue hyperperfusion followed by a sustained hypoperfusion of intestinal tissue after CA [37, 38]. This inevitably leads to a prolonged period of relative ischaemia where the intestine receives only 5% of cardiac output [39] and fosters further tissue damage. Interestingly, we found a significant correlation between the duration of CA and the Chiu-grade in the 7 d group. However, these results should be considered with caution because significant mucosal damage was also shown with a shorter duration of CA in the 6 h group (Additional file 1).

It must be noted that mucosal damage and cytokines were not analyzed in animals that could not be resuscitated. However, results were compared to a control group serving as reference. Since achievement of ROSC is mainly dependent on heart function, significant intestinal damage detectable immediately after ceasing CPR was not assumed. As reported, mucosal damage develops within the first 6 to 24 h after cardiac arrest [36] and may contribute to the development of the PCAS, which was the main objective in this investigation.

### Cytokine expression in tissue and serum

A systemic ischaemia-reperfusion-injury causes a so-called sterile inflammation, which is associated with influx of neutrophils and macrophages, leading to the production of inflammatory cytokines [40]. IL-1α, present in gastrointestinal epithelial and endothelial cells [41], acts via initiation of the inflammatory cascade and is thus a valuable parameter for the determination of inflammatory processes. In the present study, IL-1α showed a significant increase in the jejunum, resulting in the highest concentrations at 24 h post-ROSC. At the same timepoint, IL-1α concentrations in serum were close to detection limits. It has been reported that in vitro circulating IL-1α was released from endothelial cells [41] but was barely detectable in patients suffering from severe inflammation [42]. This suggests that jejunal and serum IL-1α concentrations do not necessarily match, as observed in the present study.

The IL-10 family acts protectively during intestinal inflammation by induction of anti-inflammatory effects and inhibition of pro-inflammatory cytokines such as IL-1β, IL-6 and TNF-α [43]. In our study, in jejunal tissues, IL-1α, IL-1β and TNF-α increased significantly at 24 h post-ROSC indicating that increasing IL-10 concentrations at the same time might counteract the increase of pro-inflammatory cytokines such as IL-1α, IL-1β and TNF-α.

Overall, serum cytokine concentrations were not significantly altered except for a significant increase in IL-1β after 24 h post-ROSC. Additionally, IL-1α, IL-1β, IL-10 and TNF-α concentrations were lower in serum than in the jejunum. This may indicate that the observed intestinal inflammation may not be associated with systemic inflammation.

Notably, intestinal cytokine increments were in accordance with morphological changes of the intestinal mucosa. Intestinal tissue damage peaked at 6 h post-ROSC and decreased subsequently at 24 h and 72 h. Tissue repair mechanisms accompanied by increases in intestinal inflammatory cytokine release seem to have been initiated. Consequently, desquamation of villus tips, development of a Gruenhagen’s space, hydropic generation of epithelial cells and changes in muscular layer thickness were observed.

The mechanism, which induces a further increase in intestinal tissue damage 7 d post-ROSC, accompanied by rising cytokine concentrations of IL-1β and IL-10 at the same timepoint, requires further research. As the duration between 72 h and 7 d post-ROSC is comparably long, it might be possible that further changes in cytokine concentrations remained undetected.

## Conclusions

Six minutes of transient systemic ischaemia and reperfusion resulted in mild small intestinal tissue damage but not in systemic inflammation. A rat model of 6 min CA is not capable of mimicking a PCAS. Whether there is a vital influence of the intestine on the PCAS still remains unclear and should be investigated in further studies.

## Additional file

Additional file 1: Duration of cardiac arrest in the different post-ROSC groups. One-way ANOVA *p* < 0.05. Tukey’s multiple comparison test did not show any statistically significant differences between any of the groups. (DOCX 28 kb)

## Abbreviations

ATP: Adenosine triphosphate
CA: Cardiac arrest
CPR: Cardio-pulmonary resuscitation
IL: Interleukin
NMDA: N-methyl-D-aspartate receptor
PCAS: Post cardiac arrest syndrome
ROS: Reactive oxygen species
ROSC: Return of spontaneous circulation
TNF-α: Tumor necrosis factor-α
